# Association of feeding and parenting styles with adiposity in young children: a systematic review and meta-analysis

**DOI:** 10.1007/s00431-025-06348-6

**Published:** 2025-08-04

**Authors:** Divya Nair Haridas, Prafulla Shriyan, Angham Ibrahim Tartour, Tawanda Chivese, Onno C. P. van Schayck, N. Sreekumaran Nair, Giridhara R. Babu

**Affiliations:** 1 Department of Family Medicine, Care and Public Health Research Institute (CAPHRI), Maastricht University, Maastricht, The Netherlands; 2 Department of Public Health Science, Indian Institute of Public Health Gandhinagar, Gandhinagar, Gujarat India; 3 Department of Epidemiology, Indian Institute of Public Health Bangalore, Bangalore, Karnataka India; 4 Public Health Foundation of India, New Delhi, India; 5 Department of Population Medicine, College of Medicine, QU Health, Qatar University, Doha, Qatar; 6 Department of Science and Mathematics, School of Integrated Arts and Sciences, University of Washington, Tacoma, WA USA; 7 Department of Biostatistics, Jawaharlal Institute of Postgraduate Medical Education & Research, Puducherry, India

**Keywords:** Adiposity, Feeding style, Parenting style, Children

## Abstract

This systematic review and meta-analysis synthesises global evidence on the different feeding and parenting styles, i.e., authoritative, authoritarian, indulgent, and uninvolved, and their association with adiposity in children from 6 months to 5 years of age. We searched PubMed, Ovid EMBASE, PsycINFO and Web of Science from Jan 1, 1900 to September 1, 2023, and updated the search on June 13, 2025. The primary outcome of interest was childhood adiposity. Statistical synthesis was performed using vote counting based on direction of effect, a method used for synthesizing results, by categorizing the effect of each study as beneficial or harmful based on the direction of effect. The inverse variance heterogeneity (IVhet) model, an estimator under the fixed effect model assumption with a quasi-likelihood-based variance structure, was used to conduct a meta-analysis. Of the 10,683 records screened, eight were eligible for the systematic review. These studies were conducted in high-income countries and the sample size ranged from 67 to 1238 participants. Of the eight records, three studies were eligible for meta-analysis and five studies were only eligible for vote counting based on direction of effect, as no effect estimates/95% confidence intervals (CIs) were reported. Three of the five studies synthesized using the vote counting method, found demandingness, authoritative, permissive, neglectful and uninvolved styles to be negatively associated with adiposity in children. Meta-analysis revealed the estimated effects of authoritarian (*β* = 0·02, 95% CI − 0·07 to 0·11, *I*^2^ = 22·2%, participants = 933), indulgent/permissive (*β* = 0·03, 95% CI − 0·02 to 0·26, *I*^2^ = 58·9%, participants = 872) and uninvolved/neglectful (*β* = 0·05, 95% CI − 0·005 to 0·10, *I*^2^ = 0·0%, participants = 835) styles on adiposity to be negligible. *Conclusion*: These findings suggest no consistent link between childhood adiposity and various feeding and parenting styles, including authoritarian, uninvolved, neglectful, and authoritative. Most studies had a high risk of bias, and the evidence quality was low.

**Table:** 

**What is Known:** *• To the best of our knowledge, no systematic reviews have been conducted so far focusing on the association between feeding and parenting styles and adiposity in children aged 5 years and younger.*	
**What is New:** *• This systematic review and meta-analysis of longitudinal studies conducted in high income countries found no association between feeding and parenting styles and adiposity in 6 months – 5-year-old children.* *• Methodologically robust future studies are needed to understand the causal relationship of feeding and parenting styles on adiposity in children of this age group.*

**Supplementary Information:**

The online version contains supplementary material available at 10.1007/s00431-025-06348-6.

## Introduction

Childhood obesity has emerged as a serious global health concern, with 37 million overweight children under the age of five, according to the World Health Organization (WHO) 2022 estimate [1]. This rise stems from a complex interaction of multiple factors, including genetic predisposition and environmental influences on early development [2–4]. Maternal health, formula feeding, and exposure to obesogenic settings all contribute. The persistent marketing of unhealthy foods, easy access to ultra-processed options, and reduced physical activity among youth worsen the issue [3]. The health and economic impacts of obesity necessitate addressing this epidemic. Parenting styles that combine warmth and structure are key in promoting healthy behaviours and reducing the risk of obesity in children [2].

Feeding and parenting styles are based on the combination of demandingness and responsiveness. Responsiveness involves warmth, acceptance, and participation; demandingness relates to control, maturity demands, and monitoring. In feeding, responsiveness is how parents encourage eating, while demandingness is how they motivate it. These dimensions define four main styles: authoritative, authoritarian, indulgent, and uninvolved [5–8] (Table 1). There is varying evidence on how different feeding and parenting styles affect childhood obesity. An indulgent feeding style is associated with a higher risk of childhood obesity, whereas an authoritative style tends to result in healthier weight outcomes [9, 10]. Research also links certain controlling feeding practices, such as pressuring children to eat or restricting food, to various weight statuses and emotional eating behaviours, underscoring the complexity of feeding influences on child obesity [9, 11–13]. It is important to note that caregiver feeding may be a response to a child’s weight rather than a cause. A study showed that mothers of overweight white children, concerned about their child’s weight, changed their feeding behaviour, leading to reduced weight over time [14]. Another study found greater parenting control for heavier girls [15]. Parents who perceived their children as overweight reported using less pressure during feeding, whereas those who perceived their children as lean were more likely to use pressure [16–18].

**Table 1:** Feeding and parenting styles derived from combinations of high/low demandingness and responsiveness dimensions

Responsiveness	Demandingness		
		High	Low
	High	Authoritative	Indulgent
	Low	Authoritarian	Uninvolved

Understanding the relationship between feeding and parenting styles and childhood obesity is crucial for developing effective interventions and prevention strategies. By identifying the feeding and parenting styles and practices associated with healthy eating behaviours and weight management in children, healthcare professionals and policymakers can tailor interventions to promote positive feeding environments and reduce the risk of childhood obesity.

The evidence regarding feeding and parenting styles and adiposity in children under five is underexplored and mainly derived from cross-sectional studies and many of the studies are from high-income settings [9, 19, 20]. Evidence has shown that children who are overweight during early childhood tend to remain overweight or develop obesity in adulthood [21, 22]. It is therefore important to study the 6 months to 5 years age group, as babies will start on solid food at 6 months and, since then, be slowly introduced to every food an adult in the family consumes. Studies show that children develop food preferences as preschoolers [23–27]. Parenting and feeding styles can play a crucial role in instilling the right food behaviours in the target age group, thereby preventing childhood obesity and reducing the future risk of obesity.

This systematic review and meta-analysis aimed to synthesise global evidence on different feeding and parenting styles (authoritative, authoritarian, indulgent, uninvolved) and their association with adiposity in children from 6 months to 5 years of age.

## Materials and methods

This systematic review and meta-analysis was conducted following the Preferred Reporting Items for Systematic Reviews and Meta-Analyses (PRISMA) guidelines [28]. We have published a protocol outlining the methodology (International Prospective Register of Systematic Reviews registration number: CRD42023356014) [29].

### Study eligibility — inclusion and exclusion criteria

Case–control and cohort studies on children aged 6 months–5 years with no evidence of adiposity/obesity or underweight before 6 months of age were included. Cross-sectional studies were excluded due to their inability to establish causality and the potential for reverse causality, where caregivers modify their feeding behaviour in response to the child’s weight. We included studies that considered feeding and parenting styles categorized as authoritative, authoritarian, indulgent, and uninvolved, irrespective of exposure/comparator categories and those with measures of demandingness and responsiveness as independent variables. Studies published after 1900 were included in the systematic review with no restriction on language, time or type of setting. Studies describing feeding practices such as restriction, pressure to eat, and rewarding were excluded. Since some studies found parenting styles to be associated with parental food behaviours [30, 31], in this paper, we have used both feeding and parenting styles without any distinction. Studies without an effect estimate, standard deviation, or both, and those with inconsistent effect measures across the studies were excluded from the meta-analysis.

### Data sources

We searched four electronic databases: PubMed, Ovid EMBASE, PsycINFO, and Web of Science on 01/09/2023 and updated the search on 13/06/2025. Grey literature was searched using OpenGrey and Grey Literature Report. The websites of the World Health Organization and other organisations were searched, and a review of references and co-cited articles from the selected studies was conducted.

### Search strategy

Quantitative studies were sought using the developed search strategy using year of publication filter. Search strategies are provided in Online Resource 1.

### Screening of studies

EndNote 20 was used to manage study references and remove duplicates. DNH and PS independently screened the articles in Rayyan by title and abstract to assess eligibility, followed by full-text review using the established eligibility criteria. Disagreements between the reviewers at any stage were resolved by discussing with AIT and GRB.

### Data extraction

A predesigned data extraction sheet was used to extract data on study design, country, region, data collection period, population, sample size, tool used to measure feeding/parenting style, number of items in the tool, mean age at which outcome and exposure were measured, percentage of female children, type of analysis, method of outcome standardization, outcome, length of follow-up, exposure, confounders, effect estimates, standard deviation of effect estimates and 95% confidence intervals (CIs) from the selected articles. Data was extracted by DNH and checked for accuracy by PS, GRB, AIT, and TC. Attempts were made to obtain missing data by contacting authors. Confidence intervals were computed from the reported *p*-values [32]. Any discrepancies were resolved by discussion.

### Primary outcome

The primary outcome of interest was childhood adiposity. BMI z-score, BMI percentile, BMI-for-age percentile z-score, weight status, and BMI categorical score were used as measures of childhood adiposity, obtained from the included studies.

### Risk of bias in individual studies

DNH and PS evaluated the risk of bias in the individual observational studies by using The Risk of Bias In Non-randomized Studies- of Exposure (ROBINS-E) [33]. Disagreements between the reviewers were resolved by involving AIT as the third reviewer. Robvis, a visualization tool, was used to create a risk-of-bias plot [34].

### Data synthesis

Vote counting based on direction of effect was used for statistical synthesis of studies without an effect estimate, standard deviation, or both. This method is used for synthesizing results, by categorizing the effect of each study as beneficial or harmful based on the direction of effect when only direction of effect is reported, or the effect measures or data reported across studies are inconsistent [35]. Standard deviations could not be calculated due to unavailability of relevant data for imputing standard deviation from other included studies [32]. A meta-analysis was conducted on adjusted regression coefficients with 95% CI using the inverse variance heterogeneity (IVhet) model, an estimator under the fixed effect model assumption with a quasi-likelihood-based variance structure that was found to perform better than both fixed and random effects models [36].

The ${I}^{2}$ statistic was used to quantify the heterogeneity between the studies. 25%–50% indicated low, 50%–75% stated medium, and > 75% showed high heterogeneity [37]. STATA software (version 18) was used for data analysis. The Doi plots were used considering their superiority to funnel plots when the number of studies is small [38].

### Confidence in evidence

The Grades of Recommendation, Assessment, Development, and Evaluation (GRADE) system was used to assess the quality of evidence. The domains evaluated were study design, risk of bias, degree of inconsistency, imprecision, indirectness of results, publication bias, large effect, plausible confounding, and dose–response gradient [39, 40]. Quality rating was done by DNH and PS by using GRADEpro GDT software [41]. Disagreements were resolved by discussion.

Deviations from the protocol were reported in Online Resource 2.

## Results

**Fig. 1:**
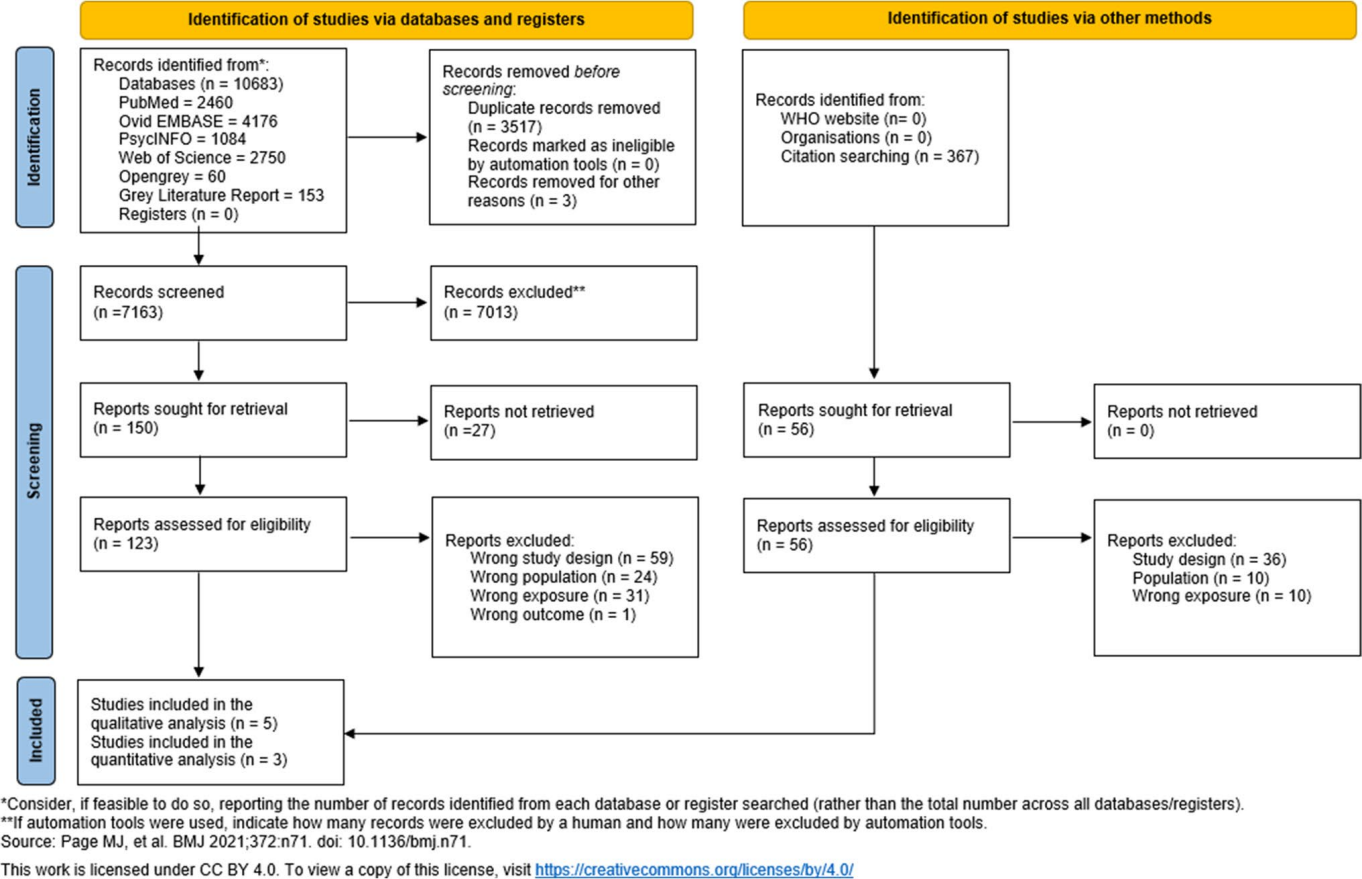
PRISMA flow diagram

A total of 10,683 records were identified through the search (Fig. 1). After duplicate removal, 7163 were screened using the title and abstract, of which 123 records were assessed for eligibility using full-text screening. Of these, 115 articles were excluded due to wrong study design (*n* = 59), wrong outcome (*n* = 1), wrong population (*n* = 24) and wrong exposure (*n* = 31). Of the eight records that met inclusion criteria, three studies were eligible for meta-analysis [42–45], and five studies were only eligible for a statistical synthesis, due to missing effect estimates/95% CIs [46–49].

### Study characteristics

Study characteristics are presented in Table 2. All eight studies were conducted in high-income countries (five—USA, two—UK, one—Australia) and used a longitudinal study design, except one with a longitudinal design followed by a cross-sectional design [45]. The sample size of the studies ranged from 67 to 1238 participants aged 0–5 years at baseline. Only two studies reported the mean age of children [45, 46]. The follow-up periods ranged from 0·5 to 11 years.

**Table 2:** Study characteristics

**Study, year**	**Design**	**Country**	**Region**	**Sample size at follow-up (excluding missing data)**	**Population**	**Mean Age (in years)**	**% female**	**Outcome**	**Method of outcome standardization**	**Tool used to measure feeding/parenting style, number of items**	**Parenting/feeding style**	**Setting**	**Length of follow-up (in years)**	**Confounders**
Chaidez, 2014 [42]	Longitudinal	USA	California-Davis	67	Toddler between the ages of 12 and 24 months	Not reported	52%	BMI z-score	WHO 2006 standards and the Centers for Disease Control growth reference	Toddler feeding questionnaire, 34	Indulgent and Authoritative scores	Not reported	0·5	Birth weight, gender, baseline z-score, maternal education, income
Fairley, 2015 [43]	Longitudinal	United Kingdom	Bradford	987	Children of 6 months of age	Not reported	Not reported	BMI z-score	WHO 2006 growth standards	Caregiver’s feeding styles questionnaire, 38	Authoritative, Authoritarian, Indulgent, Uninvolved categories	Not reported	1	Ethnicity, infant sex, maternal age, maternal highest educational qualification, parity, birthweight, gestational age at delivery, mode of delivery
Bergmeier, 2014 [46]	Prospective	Australia	Victoria	201	Children aged between 2 and 5 years	2·92	57·7%	BMI z-score	Not reported	Questionnaire, 15	Warmth and control subscales	Home	1	Maternal educational achievement, family income, maternal BMI
Connell, 2014 [47]	Longitudinal	USA	Little Rock; Irvine; Lawrence; Wellesley; Philadelphia; Pittsburgh; Morgantow; Charlottesville; Seattle; Madison	778	Children of 4 years	Not reported	52%	Age- and sex specific BMI	National reference criteria were used to calculate age- and sex-specific BMI percentiles	Not reported	Authoritative, Permissive, Neglectful, Authoritarian categories	Home, child care, school, laboratory playroom	11	Family income-to-needs ratio at age 4, mother’s education (number of years of school completed), pubertal status
Lane, 2013 [44]	Longitudinal	USA	Little Rock; Irvine; Lawrence; Wellesley; Philadelphia; Pittsburgh; Morgantow; Charlottesville; Seattle; Madison	1238	Children from birth through 11 years	Not reported	48·9%	BMI percentile	Not reported	Not clear	Authoritative, Permissive, Authoritarian, Neglectful categories	Home, child care (if used), school, laboratory playroom	11	Not reported
Perez, 2022 [48]	Longitudinal	USA	Not reported	230 dyads at 4·5-years, 212 dyads at 6-years, 240 dyads at 7·5-years	Children of 6 weeks	Not reported	54·3%	BMI-for-age percentile z-scores (BMI z%)	Calculated using World Health Organization (WHO) software for the 4·5-, 6-, and 7·5-year time points	Caregiver’s Feeding Styles Questionnaire, 19	Responsiveness and demandingness feeding dimensions	Prenatal clinics, home, lab	4·5	Prenatal maternal acculturation, infant temperamental regulation
Marshall, 2018 [49]	Longitudinal	USA	North Carolina	227	Self‐identified Latinas with a coresident child aged 2.5–3.5 years and at least one member of the household engaging in farm work during the previous year	Not reported	Not reported	Age‐ and gender‐specific BMI percentiles	Standard CDC growth charts were used to determine age‐ and gender‐specific BMI percentiles for children	Caregiver’s Feeding Style Questionnaire, 13	Demandingness and responsiveness scores	Homes or another preferred location	2	Mother’s age and education level, employment, status as a migrant versus seasonal farmworker, documentation status, mother’s weight, and time
Uerlich, 2021 [45]	Longitudinal and cross-sectional	United Kingdom	Bradford	171	Women who were between the 26th and 28th week of gestational age and their children who were born between 2007 and 2011	5·6	53·8%	BMI	The WHO reference charts for children were used to calculate the BMI	Caregiver’s Feeding Styles Questionnaire, 19	Authoritarian, Authoritative, Indulgent, Uninvolved feeding style scores	Home, school, research clinics	2·5	Not reported

### Risk of bias within studies

**Fig. 2:**
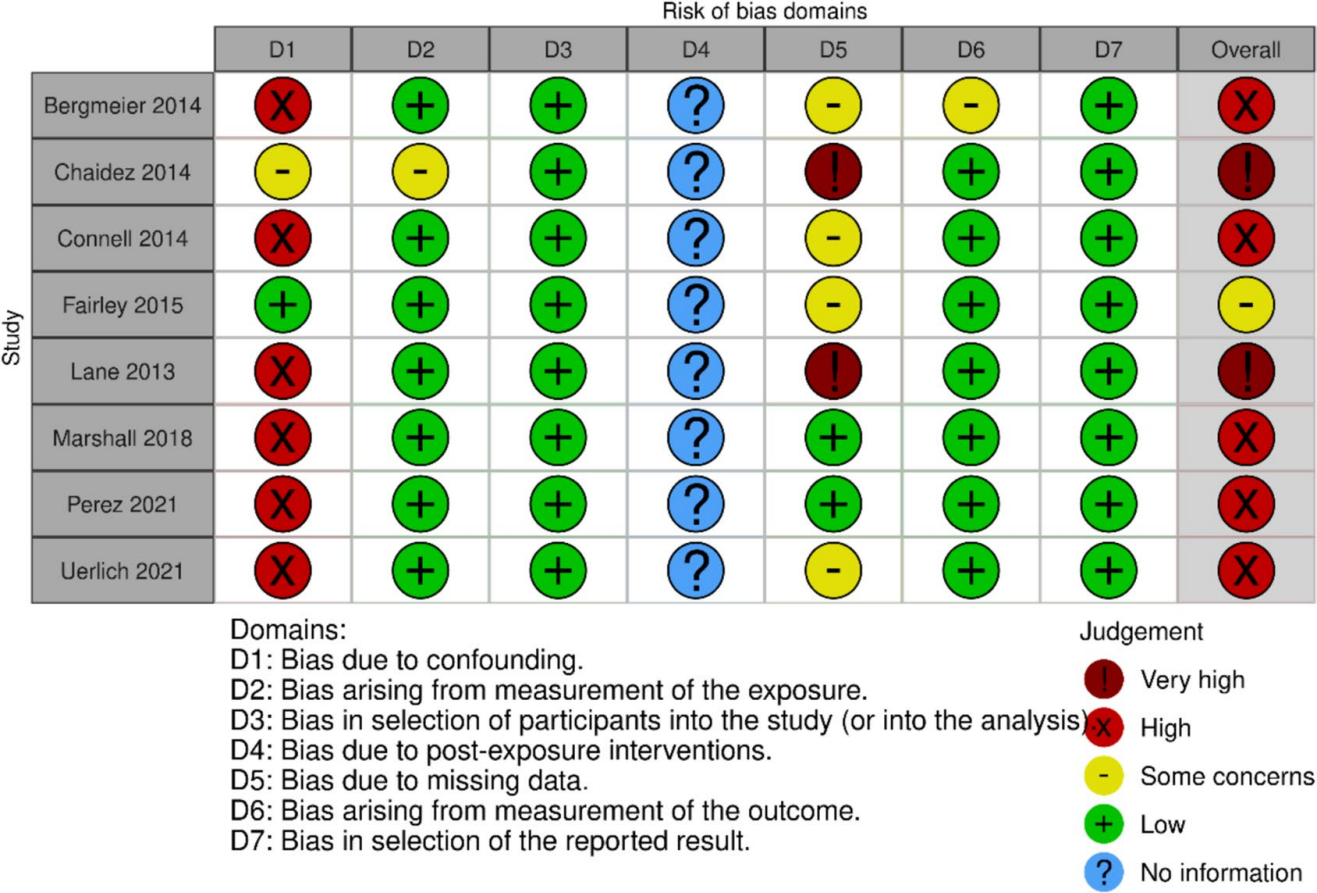
Risk of bias within studies

High risk of bias due to confounding resulted in an overall judgment of high risk of bias in the effect of exposure on outcome in five studies. These studies did not adjust for all or most of the confounders, such as child’s sex, age, only child status, birth weight, caregiver’s education and income, parent BMI, and food motivation [45–49]. Missing data with no attempt at imputation resulted in two studies having a very high risk of bias [42, 44]. Although imputed appropriately, one study had ‘some concerns’ due to incomplete data on exposure status, confounders, and outcome [43] (Fig. 2).

### Qualitative synthesis

Vote counting based on direction of effect was performed for the five studies excluded from the meta-analysis. One study found demandingness to be negatively associated with BMI-for-age percentile z-score intercept (*β* =  − 0·172, *p* < 0·05) [48] while, another found no association between BMI z-score, control (*β* =  − 0·03) and warmth (β =  − 0·02) [46]. Two studies found that children of neglectful, authoritarian mothers had higher gains in BMI than children of authoritative, permissive mothers [47] and a significant increase in the BMI categorical score with increasing feeding style score [45]. Marshall et al. demonstrated that responsiveness (*β* =  − 0.01) was independent of weight status [49]. Vote counting based on direction of effect indicated that measures of feeding and parenting styles led to adiposity in all five studies [45–49]. No graphical display was attempted, considering the small number of studies. All studies had a high risk of bias due to confounding, which reduced our confidence in the effect estimates, primarily because of insufficient adjustment for important confounders.

### Meta-analysis

All three studies that tested the association of indulgent, permissive, uninvolved, neglectful, authoritarian, authoritative feeding and parenting styles with adiposity showed no significant associations [42–44].

**Fig. 3:**
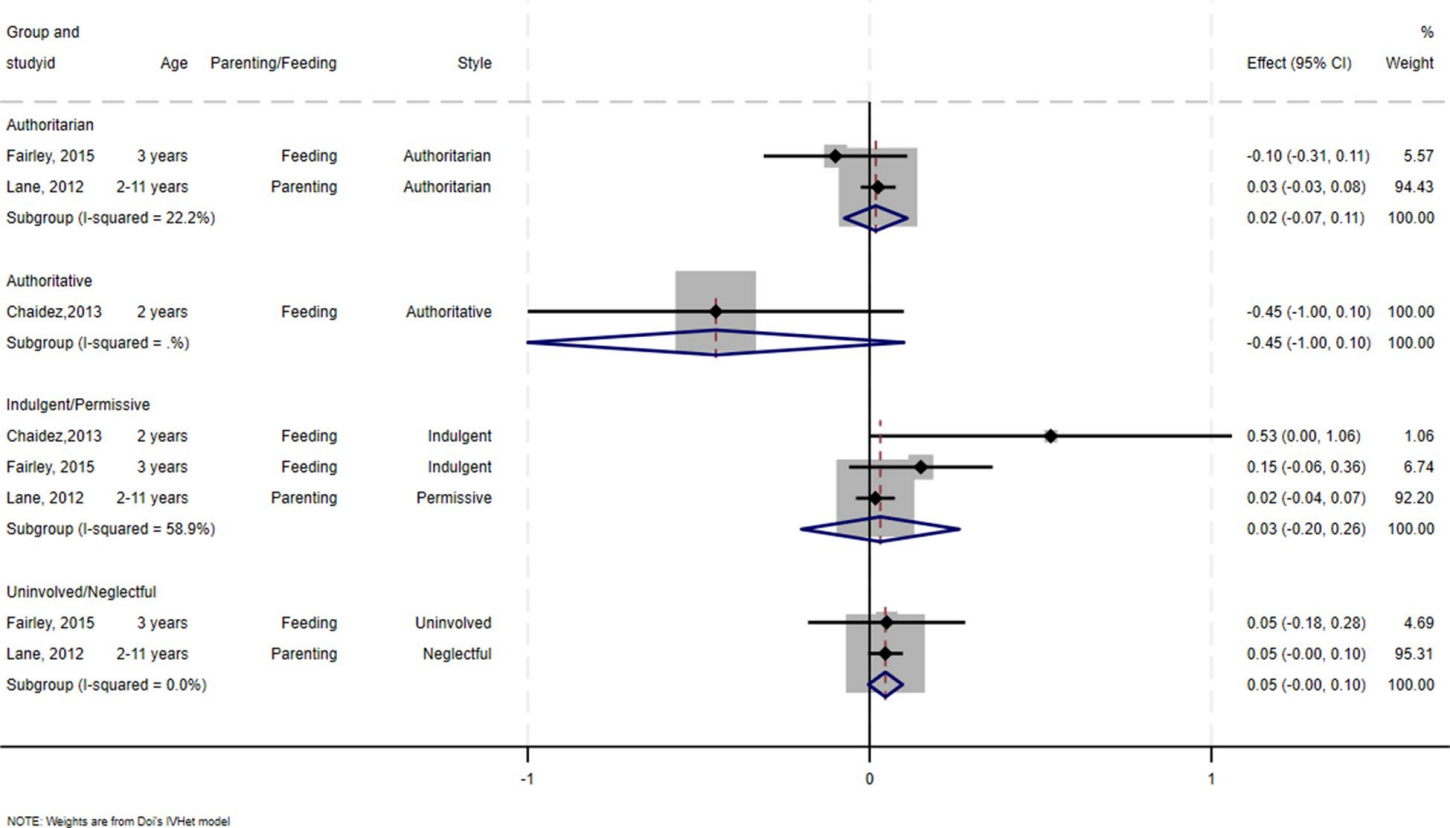
Forest plot for the effect of feeding and parenting styles on adiposity measures

Due to the small number of studies eligible for meta-analysis, we grouped permissive and indulgent styles to form the “permissive/indulgent” category and uninvolved and neglectful styles to form the “uninvolved/neglectful” category. Permissive and neglectful parenting styles were found to be conceptually equivalent to indulgent and uninvolved feeding styles, respectively [44, 50]. In the meta-analysis conducted, the estimated effect of authoritarian (*β* = 0.02, 95% CI − 0.07 to 0.11, *I*^2^ = 22.2%, participants = 933), and uninvolved/neglectful (*β* = 0.05, 95% CI − 0.005 to 0.10, *I*^2^ = 0.0%, participants = 835) styles suggested no impact on adiposity. Similarly, permissive/indulgent style (*β* = 0.03, 95% CI − 0.02 to 0.26, *I*^2^ = 58.9%, participants = 872), suggested no effect on adiposity. All three styles had a positive direction of effect. Of the three studies, two had a very high risk of bias, leading to potentially biased pooled estimates [42, 44] (Fig. 3).

The Doi plot showed no asymmetry with an LFK index of − 0.13 within the limits of ± 1. An LFK index beyond the limits of + 1 and − 1 is considered asymmetric [38] (Online Resource 3). Comparing the outcomes reported in methods and results sections of published records confirmed the absence of outcome reporting bias.

### Certainty of evidence

GRADE certainty assessment revealed very low certainty of evidence due to risk of bias, inconsistency, indirectness, imprecision, and plausible confounding (Table 3). Explanation for GRADE decisions is provided in Online Resource 4.

**Table 3:** Certainty assessment using GRADE and summary of findings

Certainty assessment							Summary of findings				Certainty
							№ of patients		Effect		
№ of studies	Study design	Risk of bias	Inconsistency	Indirectness	Imprecision	Other considerations	[intervention]	[comparison]	Relative (95% CI)	Absolute (95% CI)	
Obesity (assessed with: BMI measures)							Studies on which vote counting based on direction of effect was performed				
5	non-randomised studies	serious^a^	serious^b^	serious^c^	extremely serious^d^	publication bias strongly suspected^e^	Two studies found no association between feeding and parenting styles and adiposity. Three studies found feeding and parenting styles to be associated with obesity.				⨁◯◯◯Very low^a,b,c,d,e^
Obesity (assessed with: BMI measures)							Authoritaitve Authoritarian				
2	non-randomised studies	very serious^f^	very serious^g^	not serious^h^	not serious^i^	none^j^	320	613	-	MD 0.02 units more (0.07 fewer to 0.11 more)	⨁◯◯◯Very low^f,g,h,i,j^
Obesity (assessed with: BMI measures)							Authoritaitve Uninvolved/Neglectful				
2	non-randomised studies	very serious^f^	not serious^k^	not serious^h^	not serious^i^	none^j^	320	515	-	MD 0.05 units higher (0.005 lower to 0.1 higher)	⨁◯◯◯Very low^f,h,i,j,k^
Obesity (assessed with: BMI measures)							Authoritaitve Indulgent/Permissive				
3	non-randomised studies	very serious^l^	not serious^m^	not serious^n^	serious^o^	none^j^	320	552	-	MD 0.03 units higher (0.2 lower to 0.26 higher)	⨁◯◯◯Very low^j,l,m,n,o^
Obesity (assessed with: BMI measure)							Authoritative				
1	non-randomised studies	serious^p^	not serious^q^	not serious^r^	serious^s^	none^j^	55		-	mean 0.45 units lower (1 lower to 0.1 higher)	⨁◯◯◯Very low^j,p,q,r,s^

*CI*, confidence interval; *MD*, mean difference

## Discussion

This systematic review and meta-analysis, critically evaluated the available evidence on impact of different feeding and parenting styles on childhood adiposity. Our systematic search yielded eight studies from high-income countries. Of the five studies synthesized using vote counting based on the direction of effect, three indicated significant associations between specific feeding and parenting styles—authoritative, authoritarian, permissive, and demandingness—and adiposity measures [45–49]. In contrast, our meta-analysis did not demonstrate a statistically significant association of permissive/indulgent, neglectful/uninvolved and authoritarian styles with adiposity measures, although the trends were generally positive (Fig. 3) [42–44].

An earlier review of children aged 4–12 years found that neglecting parenting and indulgent styles associate with higher BMI and overweight risk [9]. Sleddens et al. found that authoritative parenting encourages healthy weight-related behaviours [19]. A systematic review of low-income families showed the highest BMI z-score differences among indulgent, authoritative, and authoritarian feeding styles. It found no significant difference between authoritative and authoritarian parents and child weight status [51]. None of the studies included in the meta-analysis showed a link between feeding and parenting styles and adiposity. This may be due to the specific age group, exclusion of cross-sectional studies, inadequate confounder adjustment, or missing data in included studies.

Each study examined different predictors, some appearing significant by chance. The meta-analysis clarified this, showing different feeding and parenting styles generally had no effect on child adiposity. We hypothesized that these styles are independent of time, though parents may adapt their styles based on observed feeding behaviours, making interpretation more complex than initially assumed. However, except one study [49], none of the included studies considered measuring feeding/parenting styles at multiple time points. It is also essential to consider methodological factors in studies, such as inclusion and exclusion criteria and confounders adjusted for, to ensure that observed associations are not due to reverse causation. In the systematic review of 4- to 12-year-olds, five studies found a significant link between feeding and parenting styles and child weight [9]. Of these, three studies couldn’t adjust for baseline child weight due to their cross-sectional design, though three studies adjusted for parent BMI [52–54]. Two longitudinal studies of children aged 8–12 and 4–8 years showed feeding and parenting styles linked to child weight after adjusting for initial weight [55, 56]. Only two studies considered birthweight among other confounders [42, 43].

A challenge in the review was that some studies lacked specific regression coefficients and *p*-values due to statistical insignificance. Including these values would have improved the pooled estimates and confidence in the findings. Our findings highlight the need for robust studies on how feeding and parenting styles influence childhood adiposity in ages 6 months to 5 years. This will help policymakers and healthcare providers decide if focusing on parenting behaviour change to prevent obesity is more urgent than treating obesity. Early behaviour modification helps children adapt and sustain change. Since adiposity negatively impacts child health and parents play a key role in shaping food habits through feeding styles, educating parents on choosing appropriate feeding styles is crucial [57–59]. Children up to 5 depend on caregivers, making it a good time to teach parents about healthy diets and positive eating habits. These measures can help prevent obesity in childhood and adulthood. More research is needed to understand feeding dynamics and their effects on children’s long-term health.

Our review’s main limitation is studies from high-income countries limits generalization to low- and middle-income countries (LMICs). This gap likely results from the limited number of cohort studies or the differing age groups studied. Understanding the scenario in these countries is important due to their varied financial stability and culture. A cross-sectional study found a link between authoritarian feeding style and better eating behaviours in low-income families [60]. Tekeba et al. found children from low-income countries to have higher odds of unhealthy feeding practices as compared to children from lower-middle-income countries [61]. Another study among low-income Hispanic mothers found that monitoring was negatively associated with child BMI z-scores and authoritative and indulgent feeding styles were positively related [62]. Black Afro-Caribbean used the most significant restriction and the lowest level of monitoring as compared to White German and White British. White German parents were found to use the least pressure [63]. Studies found cultures to vary in perceptions regarding overweight children, with some cultures considering heavy children as healthy children [64–66].

One of the studies in our meta-analysis had children with age exceeding 5 years and reported trajectories of BMI as outcome [44]. We could not extract data for only 6 months–5 years age group due to unavailability and limited access to raw data, but the study was included in the meta-analysis because it had relevant population and outcomes. Sensitivity analysis wasn’t possible in this regard, due to few studies per feeding and parenting style. The meta-analysis also included a study reporting feeding style on a continuous scale [42]. Our systematic review and meta-analysis is limited by the very low evidence certainty in all outcomes, requiring cautious interpretation. It may also lack power to detect small-to-moderate effects due to the small number of studies.

## Conclusion

Our study finds no consistent link between childhood adiposity and various feeding and parenting styles, including authoritarian, uninvolved, neglectful, and authoritative. Most studies had a high risk of bias, and the evidence quality was low. Future research should include well-designed cohort studies to clarify the relationship, especially from LMICs.

## Supplementary Information

Below is the link to the electronic supplementary material.

ESM 1 (PDF 124 KB)

ESM 2 (PDF 129 KB)

ESM 3 (PDF 152 KB)

ESM 4 (PDF 129 KB)

## Data Availability

No datasets were generated or analysed during the current study.

## Abbreviations

BMI: Body mass index
WHO: World health organization
CI: Confidence interval
Robvis: Risk-of-bias VISualization
PRISMA: Preferred reporting items for systematic review and meta-analysis
IVhet: Inverse variance heterogeneity
PROSPERO: Prospective register of systematic reviews
ROBINS-E: The risk of bias in non-randomized studies- of exposure
GRADE: The grades of recommendation, assessment, development, and evaluation
LMICs: Low- and middle-income countries
