# Supplementary material for: Association of feeding and parenting styles with adiposity in young children: a systematic review and meta-analysis
Source: Eur J Pediatr. 2025 Aug 4;184(8):527. doi: 10.1007/s00431-025-06348-6 (PMC12321671; doi:10.1007/s00431-025-06348-6)
Supplement: Supplementary file 1 — (PDF 124 KB) [file 431_2025_6348_MOESM1_ESM.pdf]

Article title: Association between feeding and parenting styles and adiposity in children of 6 months – 5 years of age: a systematic review and meta-analysis

Journal name: The European Journal of Pediatrics

Author names: Divya Nair Haridas, Prafulla Shriyan, Angham Ibrahim Tartour, Tawanda Chivese, Onno C.P. van Schayck, N. Sreekumaran Nair, Giridhara R. Babu

Affiliations

Department of Family Medicine, University of Maastricht, Maastricht, Netherlands

Divya Nair Haridas, Prafulla Shriyan & Onno C.P. van Schayck

Department of Public Health Science, Indian Institute of Public Health Gandhinagar, Gandhinagar, Gujarat, India; Public Health Foundation of India, New Delhi, India

Divya Nair Haridas

Public Health Foundation of India, New Delhi, India

Divya Nair Haridas & Prafulla Shriyan

Department of Epidemiology, Indian Institute of Public Health Bangalore, Bangalore, Karnataka, India

Prafulla Shriyan

Department of Population Medicine, College of Medicine, QU Health, Qatar University, Doha, Qatar

Angham Ibrahim Tartour & Giridhara R. Babu

Sciences and Mathematics, division of School of Interdisciplinary Arts and Sciences, University of Washington Tacoma

Tawanda Chivese

Department of Biostatistics, Jawaharlal Institute of Postgraduate Medical Education & Research, Puducherry, India

N. Sreekumaran Nair

Corresponding author

Correspondence to Divya Nair Haridas

Search strategy for PubMed, Ovid EMBASE, PsycINFO, and Web of Science

PubMed

(Pediatric Obesity[mesh] OR ((infant[mesh] OR "child, preschool"[mesh] OR child*[tiab] OR preschool*[tiab] OR "pre-school"[tiab] OR infant*[tiab] OR toddler*[tiab] OR kindergart*[tiab] OR pediatri*[tiab] OR paediatri*[tiab] OR boy[tiab] OR boys[tiab] OR girl*[tiab]) AND (obesity[mesh] OR "Body composition"[mesh] OR obes*[tiab] OR "body mass index"[tiab] OR BMI[tiab] OR adipos*[tiab] OR "body fat"[tiab] OR overweight[tiab] OR weight*[tiab] OR "body composition*" [tiab]))) AND (parenting[mesh] OR ((parents[mesh] OR parent*[tiab] OR carer*[tiab] OR caregiver*[tiab] OR mother*[tiab] OR maternal[tiab] OR father*[tiab] OR paternal[tiab]) AND (Authoritarianism[mesh] OR Permissiveness[mesh] OR style*[tiab] OR indulgent[tiab] OR permissive[tiab] OR authoritative[tiab] OR authoritarian[tiab] OR uninvolved[tiab] OR neglectful[tiab])))

Filters applied: from 1900/1/1 - 2025/06/13

Embase

1	exp childhood obesity/
2	(child* or preschool* or "pre-school" or infant* or toddler* or kindergart* or pediatri* or paediatri* or boy or boys or girl*) adj3 (obes* or "body mass index" or BMI or adipos* or "body fat" or overweight or weight* or "body composition*").ti,ab,kf.
3	exp obesity/
4	exp child/
5	exp child parent relation/
6	(parent* or carer* or caregiver* or mother* or maternal or father* or paternal) adj3 (style* or indulgent or permissive or authoritative or authoritarian or uninvolved or neglectful).ti,ab,kf.
7	exp parent/
8	exp authority/
9	3 and 4
10	7 and 8
11	1 or 2 or 9
12	5 or 6 or 10
13	11 and 12
14	limit 13 to to yr="2024 - 2025"

Web of Science

Number	Search
1:	TS = (infant OR "child, preschool" OR child* OR preschool* OR "pre-school" OR infant* OR toddler* OR kindergart* OR pediatri* OR paediatri* OR boy OR boys OR girl*)
2:	TS = (obesity OR obes* OR "body mass index" OR BMI OR adipos* OR "body fat" OR overweight OR weight* OR "body composition*")
3:	TS = (parent* OR carer* OR caregiver* OR mother* OR maternal OR father* OR paternal)
4:	TS = (Authoritarianism OR Permissiveness OR style* OR indulgent OR permissive OR authoritative OR authoritarian OR uninvolved OR neglectful)
5:	#1 AND #2 AND #3 AND #4<br>Publication date 1900-01-01 to 2025-06-13

APA PsycINFO

S1	MA infant OR MA "child, preschool" OR TI (child* OR preschool* OR "pre-school" OR infant* OR toddler* OR kindergart* OR pediatri* OR paediatri* OR boy OR boys OR girl*) OR AB(child* OR preschool* OR "pre-school" OR infant* OR toddler* OR kindergart* OR boy OR boys OR girl*) OR MM "Preschool Students" OR MM "Nursery School Students"
S2	MA pediatric obesity
S3	MA obesity OR MA "Body composition" OR MM "Obesity" OR MM "Body Fat" OR MM "Body Mass Index" OR MM "Body Weight" OR MM "Birth Weight" OR MM "Body Weight Cycling" OR MM "Overweight" OR MM "Underweight" OR MM "Weight Control" OR MM "Weight Gain" OR MM "Weight Loss" OR TI (obes* OR "body mass index" OR BMI OR adipos* OR "body fat" OR overweight OR weight* OR "body composition*") OR AB (obes* OR "body mass index" OR BMI OR adipos* OR "body fat" OR overweight OR weight* OR "body composition*")
S4	MA parents OR MM "Parenting" OR MM "Childrearing Practices" OR MM "Coparenting" OR MM "Parent Child Communication" OR MM "Parent Child Relations" OR MM "Parental Involvement" OR MM "Parenthood Status" OR MM "Parenting Skills" OR MM "Parenting Style" OR DE "Parenting Skills" OR MM "Parenting Style" OR MM "Authoritarian Parenting" OR MM "Authoritative Parenting" OR MM "Permissive Parenting" OR MM "Parents" OR TI (parent* OR carer* OR caregiver* OR mother* OR maternal OR father* OR paternal) OR AB (parent* OR carer* OR caregiver* OR mother* OR maternal OR father* OR paternal)
S5	MA parenting
S6	MA Authoritarianism OR MA Permissiveness OR MM "Authoritarianism" OR MM "Authoritative Parenting" OR MM "Permissive Parenting" OR TI (style* OR indulgent OR permissive OR authoritative OR authoritarian OR uninvolved OR neglectful) OR AB (style* OR indulgent OR permissive OR authoritative OR authoritarian OR uninvolved OR neglectful)
S7	S2 OR (S1 AND S3)
S8	S5 OR (S4 AND S6)
S9	S7 AND S8<br>Limiters - Publication Year: 20241001-20250613
