# Supplementary material for: Association of feeding and parenting styles with adiposity in young children: a systematic review and meta-analysis
Source: Eur J Pediatr. 2025 Aug 4;184(8):527. doi: 10.1007/s00431-025-06348-6 (PMC12321671; doi:10.1007/s00431-025-06348-6)
Supplement: Supplementary file 2 — (PDF 129 KB) [file 431_2025_6348_MOESM2_ESM.pdf]

Article title: Association between feeding and parenting styles and adiposity in children of 6 months – 5 years of age: a systematic review and meta-analysis

Journal name: The European Journal of Pediatrics

Author names: Divya Nair Haridas, Prafulla Shriyan, Angham Ibrahim Tartour, Tawanda Chivese, Onno C.P. van Schayck, N. Sreekumaran Nair, Giridhara R. Babu

Affiliations

Department of Family Medicine, University of Maastricht, Maastricht, Netherlands

Divya Nair Haridas, Prafulla Shriyan & Onno C.P. van Schayck

Department of Public Health Science, Indian Institute of Public Health Gandhinagar, Gandhinagar, Gujarat, India; Public Health Foundation of India, New Delhi, India

Divya Nair Haridas

Public Health Foundation of India, New Delhi, India

Divya Nair Haridas & Prafulla Shriyan

Department of Epidemiology, Indian Institute of Public Health Bangalore, Bangalore, Karnataka, India

Prafulla Shriyan

Department of Population Medicine, College of Medicine, QU Health, Qatar University, Doha, Qatar

Angham Ibrahim Tartour & Giridhara R. Babu

Sciences and Mathematics, division of School of Interdisciplinary Arts and Sciences, University of Washington Tacoma

Tawanda Chivese

Department of Biostatistics, Jawaharlal Institute of Postgraduate Medical Education & Research, Puducherry, India

N. Sreekumaran Nair

Corresponding author

Correspondence to Divya Nair Haridas

Deviations from the study protocol

Protocol method	Deviation from protocol method with justification
We proposed to include children who are in the age group of 6 months-5 years	We included two studies in which data were collected from children below as well as beyond 5 years. Since we could not extract data only from children less than or equal to 5 years, we had used the whole available data for the systematic review and meta-analysis<br>Type of deviation: modification
We had proposed to use fixed effects and random effects meta-analysis	We used IV-het model instead, as this method is found to perform better than the fixed and random effects models (19)<br>Type of deviation: modification
We proposed to use funnel-plots in addition to "trim and fill" method, to identify publication	We had used Doi plots instead of funnel-plots, as they are considered superior to funnel plots when the number of studies is small (reference)<br>Type of deviation: modification
We had proposed that subgroup analysis based on Low and Middle-Income countries vs. high income countries will be performed.	We were not able to perform subgroup analysis based on Low and Middle-Income countries vs. high income countries, as all the countries that finally satisfied the inclusion exclusion criteria were all from high income countries<br>Type of deviation: modification
Vote-counting was not proposed	Vote counting based on direction effect was performed for studies when only direction of effect was reported, or the effect measures or data reported across studies were inconsistent<br>Type of deviation: addition
We had proposed to exclude studies of non-English language	As restricting to English literature can cause language bias, we looked for relevant studies from among studies published in both English and non-English languages.<br>Type of deviation: modification
