# Supplementary material for: Association of feeding and parenting styles with adiposity in young children: a systematic review and meta-analysis
Source: Eur J Pediatr. 2025 Aug 4;184(8):527. doi: 10.1007/s00431-025-06348-6 (PMC12321671; doi:10.1007/s00431-025-06348-6)

Article title: Association between feeding and parenting styles and adiposity in children of 6 months – 5 years of age: a systematic review and meta-analysis

Journal name: The European Journal of Pediatrics

Author names: Divya Nair Haridas, Prafulla Shriyan, Angham Ibrahim Tartour, Tawanda Chivese, Onno C.P. van Schayck, N. Sreekumaran Nair, Giridhara R. Babu

Affiliations

Department of Family Medicine, University of Maastricht, Maastricht, Netherlands

Divya Nair Haridas, Prafulla Shriyan & Onno C.P. van Schayck

Department of Public Health Science, Indian Institute of Public Health Gandhinagar, Gandhinagar, Gujarat, India; Public Health Foundation of India, New Delhi, India

Divya Nair Haridas

Public Health Foundation of India, New Delhi, India

Divya Nair Haridas & Prafulla Shriyan

Department of Epidemiology, Indian Institute of Public Health Bangalore, Bangalore, Karnataka, India

Prafulla Shriyan

Department of Population Medicine, College of Medicine, QU Health, Qatar University, Doha, Qatar

Angham Ibrahim Tartour & Giridhara R. Babu

Sciences and Mathematics, division of School of Interdisciplinary Arts and Sciences, University of Washington Tacoma

Tawanda Chivese

Department of Biostatistics, Jawaharlal Institute of Postgraduate Medical Education & Research, Puducherry, India

N. Sreekumaran Nair

Corresponding author

Correspondence to Divya Nair Haridas

Doi plot

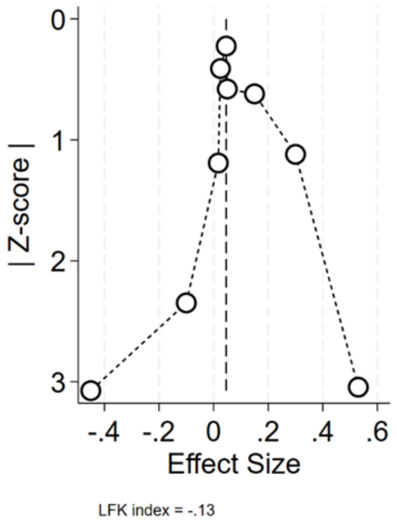

Supplement: Supplementary file 3 — (PDF 152 KB) [file 431_2025_6348_MOESM3_ESM.pdf]
