# Supplementary material for: Association of feeding and parenting styles with adiposity in young children: a systematic review and meta-analysis
Source: Eur J Pediatr. 2025 Aug 4;184(8):527. doi: 10.1007/s00431-025-06348-6 (PMC12321671; doi:10.1007/s00431-025-06348-6)
Supplement: Supplementary file 4 — (PDF 129 KB) [file 431_2025_6348_MOESM4_ESM.pdf]

Article title: Association between feeding and parenting styles and adiposity in children of 6 months – 5 years of age: a systematic review and meta-analysis

Journal name: The European Journal of Pediatrics

Author names: Divya Nair Haridas, Prafulla Shriyan, Angham Ibrahim Tartour, Tawanda Chivese, Onno C.P. van Schayck, N. Sreekumaran Nair, Giridhara R. Babu

Affiliations

Department of Family Medicine, University of Maastricht, Maastricht, Netherlands

Divya Nair Haridas, Prafulla Shriyan & Onno C.P. van Schayck

Department of Public Health Science, Indian Institute of Public Health Gandhinagar, Gandhinagar, Gujarat, India; Public Health Foundation of India, New Delhi, India

Divya Nair Haridas

Public Health Foundation of India, New Delhi, India

Divya Nair Haridas & Prafulla Shriyan

Department of Epidemiology, Indian Institute of Public Health Bangalore, Bangalore, Karnataka, India

Prafulla Shriyan

Department of Population Medicine, College of Medicine, QU Health, Qatar University, Doha, Qatar

Angham Ibrahim Tartour & Giridhara R. Babu

Sciences and Mathematics, division of School of Interdisciplinary Arts and Sciences, University of Washington Tacoma

Tawanda Chivese

Department of Biostatistics, Jawaharlal Institute of Postgraduate Medical Education & Research, Puducherry, India

N. Sreekumaran Nair

Corresponding author

Correspondence to Divya Nair Haridas

GRADE Explanation

a. Except bias due to confounding, all other domains have low or some concerns hence downgraded by 1

b. Out of 5, 3 studies showed a significant association between BMI measures and parenting and feeding styles. 2 studies did not find any association between the two measures.

c. Connell et al. did not provide estimates or statistical analyses results for evidence

d. All the 4 studies, except Uerlich et al., did not report CI and couldn't be computed due to the absence of the other required statistics

e. Selective non-reporting and under-reporting of results

f. Though one study had only some concerns because of the bias due to missing data. The other study had high bias due to confounding and very high bias due to missing data. Hence downgraded by two levels.

g. One study showed a negative direction of effect and other showed a positive direction of effect though there was no statistical significance

h. Both studies provide direct evidence for the question under consideration

i. Both studies had narrow confidence intervals and adequate sample size

j. No publication bias as suggested by Doi plot

k. Both the studies were consistent in results

l. Two studies had very high risk of bias due to missing data and one of these had high risk of bias due to confounding

m. All the studies were cosistent in results

n. All the studies provide direct evidence for the question under consideration

o. One of the studies had small sample size

p. very high risk of bias due to missing data

q. No inconsistency found

r. direct evidence for the question under consideration

s. Small sample size
